# High-Tyrosol/Hydroxytyrosol Extra Virgin Olive Oil Enhances Antioxidant Activity in Elderly Post-Myocardial Infarction Patients

**DOI:** 10.3390/antiox14070867

**Published:** 2025-07-16

**Authors:** Mojgan Morvaridzadeh, Mehdi Alami, Nada Zoubdane, Hawa Sidibé, Hicham Berrougui, Tamàs Fülöp, Michel Nguyen, Abdelouahed Khalil

**Affiliations:** 1 Geriatrics Unit, Department of Medicine, Faculty of Medicine and Health Sciences, University of Sherbrooke, Sherbrooke, QC J1H 5H3, Canada; mojgan.morvaridzadeh@usherbrooke.ca (M.M.); mehdi.alami@usherbrooke.ca (M.A.); zoubdane.nada@usherbrooke.ca (N.Z.); hawa.sidibe@usherbrooke.ca (H.S.); hicham.berrougui@usherbrooke.ca (H.B.); tamas.fulop@usherbrooke.ca (T.F.); 2 Department of Biology, Polydisciplinary Faculty, Sultan Moulay Slimane University, Beni Mellal 23000, Morocco; 3 Cardiology Unit, Department of Medicine, Faculty of Medicine and Health Sciences, University of Sherbrooke, Sherbrooke, QC J1K 2R1, Canada; michel.nguyen@usherbrooke.ca

**Keywords:** cardiovascular disease, hydroxytyrosol, extra virgin olive oil, oxidative stress

## Abstract

Cardiovascular disease (CVD), particularly atherosclerotic cardiovascular disease (ASCVD), is the leading cause of death worldwide, driven by factors like oxidative stress, inflammation, and lipid metabolism disorders. Although phenolic compounds such as Tyrosol (Tyr) and Hydroxytyrosol (HTyr) found in extra virgin olive oil (EVOO) have shown promising antioxidant and anti-inflammatory effects, their specific roles in modulating oxidative stress biomarkers and high-density lipoprotein (HDL) functionality in elderly populations, especially in those with prior myocardial infarction, are not fully understood. This study aimed to investigate the effects of EVOO phenolic compounds on oxidative stress biomarkers and HDL functionality, and related metabolic outcomes in both healthy and post-myocardial infarction (post-MI) elderly individuals. This pilot randomized clinical trial study included healthy and post-MI participants aged 65–85 years. Participants in each group were randomly assigned to consume 25 mL per day of one of three types of olive oils: high phenolic (HTyr/Tyr) extra virgin olive oil (HP-EVOO), extra virgin olive oil (EVOO), or refined olive oil (ROO) for a period of 26 weeks. Blood samples were collected at baseline and post-intervention to assess key biomarkers. Plasma levels of (poly)phenols, malondialdehyde (MDA), total antioxidant capacity (FRAP), lecithin-cholesterol acyltransferase activity (LCAT), and serum paraoxonase-1 (PON-1) activity were measured. A total of 34 individuals completed the study (mean age: 74 years). Baseline characteristics, including sex, age, body mass index (BMI), weight, blood pressure, and inflammatory markers like C-reactive protein (CRP) levels, did not differ significantly between the two groups. A significant increase in both FRAP levels and PON-1 activity was observed in post-MI participants following HP-EVOO consumption compared to baseline (*p* = 0.014). No significant changes were observed in MDA levels, LCAT activity, or plasma (poly)phenols. These results indicate that HP-EVOO may enhance antioxidant capacity, particularly FRAP and PON-1 activity, in elderly post-MI individuals. The observed differences between groups suggest that underlying cardiometabolic status may influence the response to olive oil phenolic compounds. Further studies are needed to explore the long-term cardiovascular effects.

## 1. Introduction

Cardiovascular disease (CVD) is the major cause of mortality and morbidity, affecting 523 million people worldwide. Atherosclerotic cardiovascular diseases (ASCVDs), particularly ischemic heart disease (IHD) and stroke, are the most prominent contributors to the global burden of CVD and determine variations and trends in age-standardised prevalence [1]. Atherosclerosis is a progressive condition characterized by the buildup of lipid plaques within vessel walls [2]. This process involves lipid deposition, endothelial dysfunction, oxidative stress, and immune activation, ultimately leading to plaque formation and, in advanced stages, thrombosis [3].

Several risk factors, including a sub-optimal diet, physical inactivity, smoking, and alcohol consumption, contribute to ASCVD. Evidence from multiple studies indicates that addressing these lifestyle factors can significantly reduce the risk of cardiovascular events [4], including dietary changes, increased physical activity, cessation of smoking, and alcohol consumption. Moreover, antioxidant, anti-inflammatory, and lipid-lowering therapies are among the key strategies for both the prevention and management of ASCVDs [5].

A longitudinal study of 1180 individuals with a history of ASCVD found that adherence to the Mediterranean diet (MD) significantly reduced the risk of all-cause, cardiovascular, and coronary artery disease/cerebrovascular mortality, regardless of statin use [6]. Furthermore, the combination of statin therapy and average-to-high adherence to MD showed a synergistic effect in reducing cardiovascular mortality, likely mediated by reduction in low-grade inflammation rather than changes in blood cholesterol levels [6]. It is notable that the main source of fat in MD is primarily extra-virgin olive oil (EVOO). The antiatherogenic effects of EVOO were originally attributed to its high content of monounsaturated fats (MUFA), particularly oleic acid [7]. However, recent studies indicate that the bioactive phenolic compounds present in EVOO also contribute substantially to its cardioprotective properties [8]. A controlled, crossover, randomized trial by Covas et al., including 200 healthy male volunteers from five European countries, demonstrated that olive oil with higher phenolic content significantly improved plasma lipid levels and reduced oxidative stress markers, such as oxidized low-density lipoprotein (ox-LDL), in a dose-dependent manner [9]. These findings indicate that, apart from its monounsaturated fat content, the phenolic compounds in EVOO provide cardiovascular benefits, including increasing high-density lipoprotein (HDL) cholesterol and reducing oxidative damage [9]. Similarly, Perrone et al., showed that an acute postprandial intake of 25 g phenol-rich EVOO significantly reduced oxidative stress biomarkers, such as ox-LDL and malondialdehyde (MDA), and also increased the expression of antioxidant genes, including catalase and superoxide dismutase-1 (SOD-1) in healthy participants [10].

Tyrosol (Tyr) and its hydroxylated derivative, Hydroxytyrosol (HTyr), are two phenolic compounds found in EVOO that exhibit a range of bioactive properties, including anti-inflammatory, anti-proliferative effects, and potent antioxidant activities [11]. Furthermore, Tyr and HTyr are the only phenolic compounds approved by the European Food Safety Authority (EFSA) with a health claim, supporting their ability to protect LDL from oxidative damage while also modulating HDL levels and blood pressure [12]. For optimal health benefits, the EFSA recommends a daily intake of 20 g of EVOO containing at least 5 mg of Tyr/HTyr and their derivatives [13]. The diverse biological activities of Tyr/HTyr were initially attributed to their potent antioxidant properties, primarily through free radical-scavenging and metal-chelating mechanisms [14]. These antioxidant effects stem from their hydroxyl group, which donates electrons, forms stable hydrogen bonds with phenoxyl radicals, and scavenges reactive oxygen species (ROS) [15,16]. HTyr upregulates heme oxygenase-1 (HO-1) in VECs via PI3K/Akt and ERK1/2 signaling pathways. This leads to the stabilization of nuclear factor erythroid 2-related factor 2 (Nrf2), a key transcription factor that regulates antioxidant responses. Through these effects, HTyr contributes to wound healing and may protect against atherosclerosis by mitigating oxidative stress and improving endothelial function [17].

Similarly, studies have shown that Tyr can activate the Nrf2/HO-1 pathway, enhancing the expression of antioxidant enzymes and providing cytoprotective effects. For instance, Wang et al., demonstrated that Tyr significantly upregulated HO-1 expression and activated Nrf2 in RAW 264.7 murine macrophages. Moreover, in a mouse model of lipopolysaccharide (LPS)-induced acute lung injury, Tyr treatment improved survival, reduced lung permeability, and ameliorated histopathological damage, indicating its potential protective effects against oxidative stress and inflammation [18]. Additionally, some studies have shown that HTyr lowers ROS and MDA levels while enhancing nitric oxide (NO) production in vitro and in vivo [19]. Although HTyr has been shown to exert a wide range of biological effects, including cardioprotective, endocrine, metabolic, and other health-promoting properties [20], the evidence regarding the impact of EVOO or its phenolic compounds, like Tyr/HTyr, on oxidative stress, inflammatory markers, and cardiometabolic risk factors remains inconsistent across systematic reviews and meta-analyses [21,22,23]. Conversely, inflammation has been shown to induce multiple structural modifications in HDL, leading to functional impairments [24]. In addition, inflammatory biomarkers such as C-reactive protein (CRP) are known to increase significantly following acute MI [25]. CRP levels typically rise within 4 to 6 h after symptom onset, peak between 2 and 4 days, and gradually return to baseline within 7 to 10 days [25]. However, the significance of elevated inflammatory markers during the convalescence period remains underexplored. Additionally, it has been reported that HDL maturation and functionality are compromised in post-MI patients, as evidenced by reduced cholesterol efflux from macrophages, decreased paraoxonase-1 (PON-1) activity [26], and reduced anti-inflammatory properties, independent of plasma HDL cholesterol levels [27]. Moreover, Guerin et al., highlighted that serum cholesterol efflux capacity serves as a valuable biomarker for identifying patients at higher risk of mortality following an acute coronary event. These findings suggest that enhancing serum cholesterol efflux capacity in post-MI patients may contribute to a reduction in mortality risk. Given that elderly individuals with a history of MI face elevated rates of recurrent cardiovascular events, including multiple recurrences [28], there is a clear need for intensive secondary prevention strategies and comprehensive management of comorbidities in high-risk patients. Therefore, the present study aimed to evaluate the long-term effect of EVOO enriched in phenolic compounds, particularly Tyr/HTy, on oxidative stress biomarkers in post-MI patients, in comparison to age-matched elderly individuals without a history of cardiovascular disease or other major chronic illnesses.

## 2. Materials and Methods

### 2.1. Study Design and Subjects

This study is part of the LIPIMAGE Cohort, an ongoing prospective study in which positron emission tomography imaging is used to analyze the effect of EVOO on the atherosclerotic plaque progression and stability in individuals at high cardiovascular risk. The study protocol was approved in accordance with the Declaration of Helsinki and approved by the Sherbrooke University Hospital Center Ethics Committee (#2019-3145). All participants provided written informed consent prior to enrollment.

**Figure 1:**
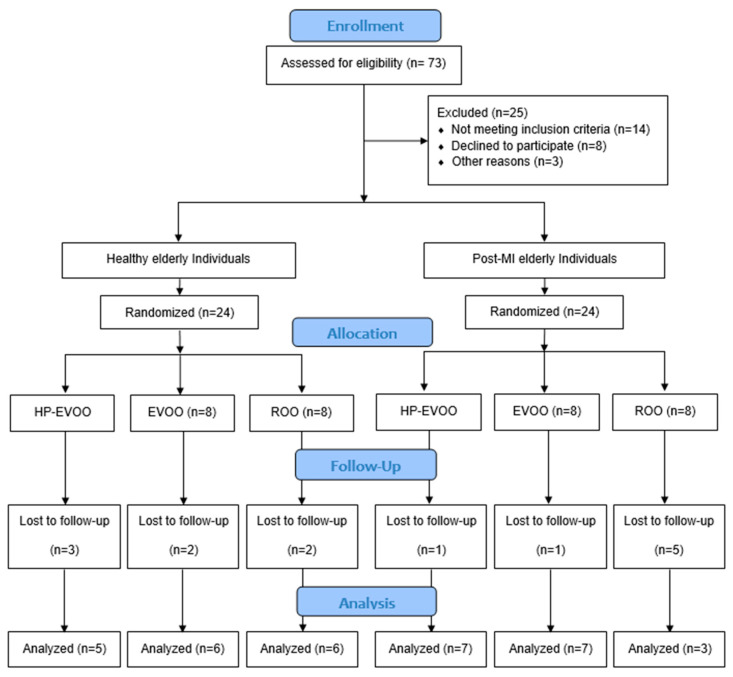
Flow diagram of study design and participant allocation.

Forty-eight participants were recruited for the study, including healthy individuals (*n* = 24) and post-MI patients (*n* = 24) who were enrolled at least three months after their infarction event to allow stabilization of infarct-related inflammation. Within each group, participants were randomized into one of three subgroups to receive one of three types of olive oils: high Tyr/HTyr-EVOO (HP-EVOO), regular EVOO, or refined olive oil (ROO). All participants consumed 25 mL per day for a duration of 26 weeks (Figure 1). The dose of 25 mL/day was selected based on previous clinical trials demonstrating its efficacy in improving cardiovascular and oxidative stress markers [29]. Healthy individuals were selected based on the absence of recent or family history of disease and to meet criteria for presenting normal arterial pressure (less than 140/85 mmHg), normal lipid profile, and normal ECG.

All participants had a BMI between 23 and 33 kg/m^2^. Exclusion criteria included high alcohol consumption, smoking, chronic metabolic disorders such as diabetes (HbA1c > 6%), chronic inflammatory conditions, liver or kidney failure, cancer, use of anti-inflammatory medications, or intake of dietary supplements such as omega-3 fatty acids, probiotics, or hormonal therapy. Post-MI patients have maintained their prescribed medications. Also, subjects consuming EVOO regularly (more than 3 times per week) were excluded.

Fasting blood samples were collected at baseline and after 6 months of olive oil consumption. During the intervention period, participants attended monthly visits to receive a new bottle of olive oil, monitor compliance, address any questions, and collect study-related data.

Participants were instructed to maintain their usual dietary habits and physical activity level throughout the six-month intervention. Dietary assessment was performed using a three-day food journal completed by each participant.

### 2.2. Olive Oils

HP-EVOO and regular EVOO were obtained from Atlas Olive Oil Inc. (Casablanca, Morocco). The HP-EVOO contained exceptionally high levels of phenolic compounds (1249 mg/kg), with particularly elevated concentrations of Tyr and HTyr, measured at 123.1 mg/kg and 233.6 mg/kg, respectively. In comparison, regular EVOO contains 255 mg/kg of total phenolic compounds, with Tyr and HTyr levels of 6.3 mg/kg and 7.8 mg/kg, respectively. The phenolic content of all oils was certified by an independent Biotechnology Laboratory at the Faculty of Sciences Dhar El Mehraz (Fez, Morocco) and cross-verified in our laboratory. ROO was purchased from local grocery stores in Sherbrooke, Canada, and was confirmed to be devoid of phenolic compounds. ROO was used as the control group for comparison with the two intervention groups receiving EVOO and HP-EVOO. ROO was selected because it contains minimal phenolic compounds, making it a suitable comparator to isolate the effects of phenolic content.

All participants were instructed to consume 25 mL of their assigned oil daily, in its raw form and preferably with meals. Compliance with the intervention was monitored during monthly visits through the administration of a standardized questionnaire. Adherence was further evaluated by collecting and analyzing the returned EVOO containers.

### 2.3. Blood Collection

Participants’ blood samples were collected following an overnight fast (at least 8 h). Blood samples were collected in EDTA tubes and promptly centrifuged at 400× *g* for 15 min to separate the plasma. Plasma and serum samples were aliquoted and stored at −80 °C until further analysis.

### 2.4. Biochemical Analyses

#### 2.4.1. Plasma Total (Poly)phenols Measurement

Plasma total (poly)phenols were measured as previously described [30], with some modifications. Briefly, 100 µL of plasma was added to 200 µL of 1 mol/L HCl, vortexed for 1 min, and incubated at 37 °C for 30 min. Then 200 µL of 2 mol/L NaOH in 75% methanol was added, followed by vortexing for 2 min and incubation at 37 °C for an additional 30 min. Next, 200 µL of 0.75 mol/L meta-phosphoric acid was added, and the mixture was vortexed for 2 min before being centrifuged at 1700× *g* for 10 min at 4 °C. The supernatant was collected and stored on ice in the dark. Residual (poly)phenols in the pellet were re-extracted by adding 200 µL of 1:1 (*v*/*v*) acetone:water. This mixture was vortexed for 1 min and centrifuged at 1700× *g* for 10 min at 4 °C to obtain the supernatant. The two supernatants were pooled together and centrifuged at 1700× *g* for 5 min at 4 °C. The ultimate supernatant was obtained after centrifugation were used for analysis. For quantification of (poly)phenols, 250 µL of 0.2 N Folin-Ciocalteu phenol reagent and 200 µL of 2 mol/L Na_2_CO_3_ solution were added to 50 µL of the final supernatant. After 90 min of incubation at room temperature in the dark, absorbance at 750 nm was measured using the Multimode Plate Reader (VICTOR^TM^ X5, PerkinElmer, Waltham, MA, USA) at the Sherbrooke University, Sherbrooke, Canada. A calibration curve was generated using Gallic acid (GA) standard, ranging from 0 to 800 µg/mL, prepared in distilled water. Results were expressed as µg of Gallic acid equivalents (GAE) per mL of plasma.

#### 2.4.2. Ferric Reducing Antioxidant Power (FRAP)

The Ferric Reducing Antioxidant Power (FRAP) of serum samples was measured using the FRAP Assay Kit (MAK509, Sigma-Aldrich, St. Louis, MO, USA) according to the manufacturer’s instructions. This colorimetric assay quantifies total antioxidant capacity by evaluating the ability of the sample to reduce ferric (Fe^3+^) to ferrous (Fe^2+^) iron in the presence of a chromogenic substrate, forming a blue-colored Fe^2+^-complex measurable at 590 nm. Briefly, serum samples were first diluted 1:10 with purified water, and all standards and samples were run in duplicate. A standard curve was generated using serial dilutions of a 180 μM Fe^2+^ solution. Fresh working reagent was prepared prior to each assay. For analysis, 50 μL of each sample or standard was added to a clear, flat-bottom 96-well plate, followed by the addition of 200 μL of the working reagent. After gentle mixing and a 40 min incubation at room temperature, absorbance was measured at 590 nm using a VICTOR™ X5 Multimode Plate Reader (PerkinElmer, Waltham, MA, USA).

FRAP values were calculated from the slope of the standard curve and expressed as μM Fe^2+^ equivalents, using the following formula:

$$FRAP=\frac{R\;\;Sample-R\;\;Blank}{Slope}\;\times \;Dilution\;Factor$$

R Sample and R Blank are the optical density values of the sample and blank, respectively. Results are expressed in μM Fe^2+^ equivalent.

#### 2.4.3. PON-1 Activity Measurement 

Serum paraoxonase activity was measured using paraoxon as substrate as previously described [31]. Briefly, 25 µL of serum was added to Tris/HCl buffer (100 mmol/L, pH 8.0) containing 2 mmol/L CaCl_2_ and 5.5 mmol/L paraoxon (*O,O*-diethyl-*O-p*-nitrophenylphosphate; Sigma Chemical Co., St. Louis, MO, USA). The rate of hydrolysis of paraoxon and generation of p-nitrophenol was determined by monitoring the increase in absorbance at 412 nm and at 25 °C (UH5300, UV/VIS spectrophotometer, Hitachi High-Tech America, Inc., Tokyo, Japan) (serial No. 3J1-0012). The amount of p-nitrophenol generated was calculated using the molar extinction coefficients at pH 8 (ε = 17,100 M^−1^ cm^−1^). Paraoxonase activity was expressed as units per milliliter (U/mL).

#### 2.4.4. MDA Measurement

Plasma MDA levels were measured by using the thiobarbituric acid reactive substances (TBARS) assay, as previously described [32,33], with minor modifications. Briefly, 75 µL of plasma was mixed with 1.5 mL TCA-TBA-HCl reagent (15% (*w*/*v*) TCA, 0.385% (*w*/*v*) TBA in 0.25 M HCl), then heated at 100 °C for 30 min in a dry bath incubator (Fisher Scientific, Waltham, MA, USA). After cooling, samples were centrifuged at 3000 rpm for 10 min at 4 °C to remove precipitates. The absorbance of MDA in the supernatant was measured at 532 nm using a VICTOR^TM^ X5 Multimode Plate Reader. Results were expressed in μmol/L, with 1,1,3,3-Tetraethoxypropane (TEP) used as the MDA standard.

#### 2.4.5. LCAT Activity Measurement

Plasma LCAT activity level was measured by using a commercially available kit (Roar Biomedical Inc., New York, NY, USA). Plasma samples were incubated with a fluorescent substrate, and the fluorescence intensity of the intact substrate was measured at 470 nm by a Bio-Tek Synergy HT MultiMode Microplate Reader (BioTek Instruments, Winooski, VT, USA). As the substrate is hydrolyzed by LCAT, a monomer is produced, which becomes detectable at 390 nm. LCAT activity was calculated by measuring the 390/470 nm emission intensity.

### 2.5. Statistical Analysis

The obtained results were reported as mean ± standard error (SE). The normality of the distribution of quantitative variables was assessed using the Shapiro–Wilk test. For within-group comparisons (pre- and post-intervention), a paired *t*-test was used for normally distributed variables, while the Wilcoxon signed-rank test was applied for non-normally distributed data. For between-group comparisons, an unpaired *t*-test was used when the data followed a normal distribution, and the Mann–Whitney U test was used for non-normal distributions. Sex distribution across the six study groups was compared using Fisher’s exact test. To compare means among the three olive oil subgroups (before and after the intervention), one-way ANOVA was used for normally distributed data, and the Kruskal–Wallis test for non-normal data. A *p*-value less than or equal to 0.05 was considered statistically significant. All statistical analyses were performed using GraphPad Prism version 10.1.2 (GraphPad Software^®^, Inc., La Jolla, CA, USA).

## 3. Results

### 3.1. Participants’ Characteristics

Of the 73 participants assessed for eligibility, 48 were enrolled in the study, and 34 completed the intervention (see Figure 1 for details). Baseline characteristics of the participants are presented in Table 1. Overall, 64% of participants were male. Sex distribution across the six groups was compared using Fisher’s exact test. No significant differences were observed (*p* = 0.438). The study groups were generally well-matched, with no statistically significant differences in mean age (healthy individuals: 74.76 ± 1.43 years; post-MI: 73.62 ± 1.56 years, *p* = 0.592), body weight, BMI, systolic blood pressure, CRP, triglycerides (TGs) and lipoprotein(a) (Lp(a)). However, a significant difference in baseline diastolic blood pressure was observed in the HP-EVOO group between healthy and post-MI participants (*p* = 0.024). Additionally, baseline lipid profiles revealed significant differences between groups in total cholesterol (TC) (*p* < 0.001), HDL-C (*p* = 0.031), LDL-C (*p* = 0.010), and non-HDL cholesterol (*p* = 0.035). These differences remained significant following the intervention for total cholesterol (TC) (*p* = 0.003), HDL-C (*p* = 0.048), LDL-C (*p* < 0.007), and non-HDL cholesterol (*p* = 0.016).

In the EVOO group, both at baseline and after the 6-month intervention, significant differences were observed between healthy and post-MI patients in TC (*p* < 0.001 and *p* < 0.0001), LDL-C (*p* < 0.001 and *p* < 0.0001), and non-HDL cholesterol (*p* = 0.002 and *p* < 0.0001).

For the ROO group, both within-group and between-group analyses revealed significant differences in LDL-C (*p* = 0.006 at baseline and *p* = 0.030 at the end of the study) and non-HDL cholesterol (*p* = 0.002 at baseline and *p* = 0.022 at the end of the study).

### 3.2. Biochemical Outcomes

**Figure 2:**
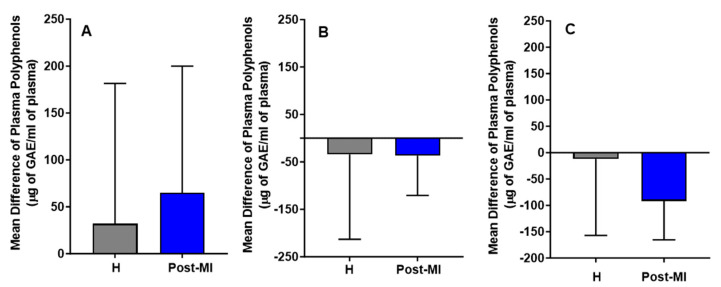
Mean differences compared to baseline in plasma (poly)phenols (µg GAE/mL) after six months of intervention with three types of oils. (**A**): high polyphenol extra virgin olive oil (HP-EVOO), (**B**): extra virgin olive oil (EVOO), and (**C**): refined olive oil (ROO), in healthy (H) and post-myocardial infarction (post-MI) participants.

Following the intervention, in the HP-EVOO group, plasma (poly)phenol levels increased in both healthy (mean difference compared to baseline = 32 ± 149 μg of GAE/mL) and post-MI participants (mean difference compared to baseline = 65 ± 134.87 μg of GAE/mL). In contrast, decreases were observed in the EVOO group (H: mean difference = −34 ± 179; PMI: mean difference = −37 ± 83.5 μg of GAE/mL) and ROO group (H: MD = −12 ± 144.9; PMI: MD = −92 ± 73.4 μg of GAE/mL). While these trends are illustrated in Figure 2, none of the changes were statistically significant, either within or between healthy participants and post-MI patients across the three intervention groups (HP-EVOO, EVOO, and ROO) (Figure 2).

**Figure 3:**
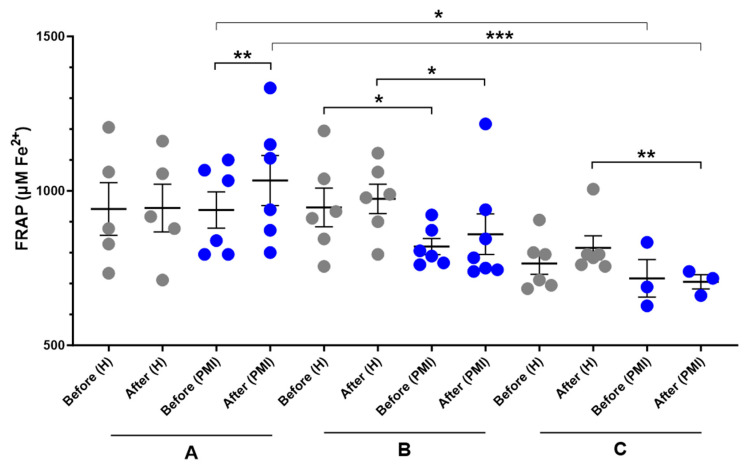
Serum FRAP (µM Fe^2+^) levels before and after six months of intervention with three types of oils. (**A**): high polyphenol extra virgin olive oil (HP-EVOO), (**B**): extra virgin olive oil (EVOO), and (**C**): refined olive oil (ROO), in healthy (H) and post-myocardial infarction (post-MI) participants. *** *p* < 0.01, ** *p* < 0.02, * *p* ≤ 0.05.

A significant increase in serum FRAP was observed in post-MI patients following HP-EVOO consumption (mean difference compared to baseline = 67.7 ± 89.9 μM Fe^2+^, *p* = 0.014) compared to the baseline, while it was small and insignificant in healthy participants (mean difference compared to baseline = 3.3 ± 114.7 μM Fe^2+^). Additionally, an increase in the mean difference was observed in both healthy and post-MI participants after consuming EVOO; however, it was not statistically significant (H: mean difference compared to baseline = 27.8 ± 78.7 μM Fe^2+^; PMI: mean difference compared to baseline = 26.9 ± 80.2 μM Fe^2+^). Moreover, results showed a significant difference between healthy and post-MI patients at baseline (*p* = 0.046) and after consuming EVOO (*p* = 0.050). In the ROO group, there was no significant difference between baseline levels of FRAP between healthy and post-MI participants, however, after the intervention, the FRAP level decreased in post-MI patients (mean difference compared to baseline = −11.1 ± 65.2, *p* = 0.427) and increased in healthy participants (mean difference compared to baseline = 50.9 ± 52, *p* = 0.062) and made the difference statistically significant (*p* = 0.011). One-way ANOVA revealed a significant difference in baseline FRAP levels between the HP-EVOO and ROO groups among post-MI patients (*p* = 0.037), and the Kruskal–Wallis test showed a significant difference in post-consumption values between these groups (*p* = 0.009) (Figure 3).

**Figure 4:**
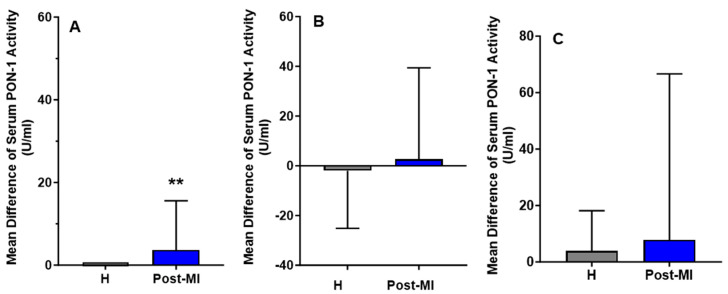
Mean differences compared to baseline of serum PON-1 activity (U/mL) after six months of intervention with three types of olive oils. (**A**): high polyphenol extra virgin olive oil (HP-EVOO), (**B**): extra virgin olive oil (EVOO), and (**C**): refined olive oil (ROO), in healthy (H) and post-myocardial infarction (post-MI) participants. ** *p* < 0.02.

For the HP-EVOO group, serum PON-1 activity was significantly higher in healthy (mean: 92.84 ± 27.53 U/mL) participants compared to post-MI patients (mean: 33.86 ± 8.55 U/mL), both at baseline (*p* = 0.020) and after the intervention (healthy participants mean: 92.84 ± 26.05 U/mL, post-MI patients mean: 37.54 ± 8.27 U/mL, *p* = 0.021). Interestingly, following 6 months of HP-EVOO consumption, serum PON-1 activity exhibited a significant increase in post-MI patients compared to baseline (PMI: mean difference compared to baseline = 3.68 ± 11.90 U/mL, *p* = 0.014) (Figure 4).

Results from the EVOO and ROO groups showed no significant changes in PON-1 activity among healthy participants, and a slight increase in post-MI patients (mean difference compared to baseline = 2.63 ± 36.78 U/mL); however, these changes were not statistically significant.

**Figure 5:**
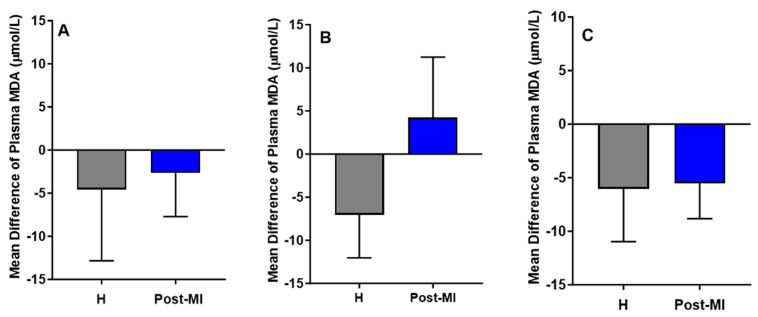
Mean differences compared to baseline of plasma MDA levels (µmol/L) after six months of intervention with three types of oils. (**A**): high polyphenol extra virgin olive oil (HP-EVOO), (**B**): extra virgin olive oil (EVOO), and (**C**): refined olive oil (ROO), in healthy (H) and post-myocardial infarction (post-MI) participants.

MDA levels decreased slightly in both healthy (mean difference compared to baseline = −4.56 ± 8.27 μmol/L) and post-MI participants (mean difference = −2.66 ± 5.05 μmol/L) in the HP-EVOO group. A similar downward trend was observed in the ROO group for both healthy individuals (mean difference compared to baseline = −6.05 ± 4.91 μmol/L, *p* = 0.055) and post-MI individuals (mean difference compared to baseline = −5.50 ± 3.33, *p* = 0.059). However, in the EVOO group, results showed a reduction in MDA levels among healthy participants (mean difference compared to baseline = −7.05 ± 4.99 μmol/L), and an increase among post-MI individuals (mean difference compared to baseline = 4.22 ± 7.02 μmol/L). Despite these trends, none of the changes were statistically significant (Figure 5). One-way ANOVA and Kruskal–Wallis tests showed no significant differences in plasma MDA among healthy participants across the three oil groups, nor among post-MI participants.

**Figure 6:**
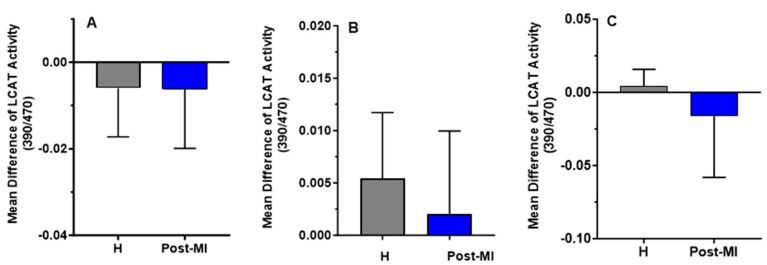
Mean differences compared to baseline of plasma LCAT activity (expressed as the 390/470 ratio) after six months of intervention with three types of oils. (**A**): high polyphenol extra virgin olive oil (HP-EVOO), (**B**): extra virgin olive oil (EVOO), and (**C**): refined olive oil (ROO), in healthy (H) and post-myocardial infarction (post-MI) participants.

LCAT activity slightly decreased after 6 months of HP-EVOO consumption in both healthy (mean difference compared to baseline = −0.006 ± 0.011) and post-MI participants (mean difference compared to baseline = −0.0062 ± 0.0137), as well as after ROO consumption in post-MI patients (mean difference compared to baseline = −0.0163 ± 0.0419). In contrast, an increase in LCAT activity was observed in both healthy (mean difference compared to baseline = 0.0054 ± 0.0063) and post-MI participants (mean difference compared to baseline = 0.002 ± 0.008, *p* = 0.332) following EVOO intake, and in healthy participants (mean difference = 0.004 ± 0.012) only after ROO intake. However, these changes were not statistically significant (Figure 6).

## 4. Discussion

In this 26-week pilot randomized clinical trial, we investigated the effects of consuming 25 mL/day of three types of olive oil on oxidative stress biomarkers and HDL functionality. Our results showed a significant increase in serum FRAP and PON-1 activity in post-MI patients (aged 65–85) after consuming 25 mL/day of HP-EVOO, compared to baseline. No adverse effects were observed. These findings suggest that incorporating 25 mL/day of HP-EVOO into the daily diet may enhance HDL functionality, particularly its antioxidant activity, and may also improve the plasma total antioxidant capacity in patients with previous MI.

Participants in both groups received supplementation with three types of olive oil over a period of six months. The daily intake of 25 mL of each oil, in our conditions, corresponded to a daily intake of 2.70 mg Tyr and 5.30 mg HTyr for the HP-EVOO, 0.14 mg and 0.18 mg, respectively, for the standard EVOO, and undetectable levels of these phenolic compounds for the ROO. The total intake of phenolic compounds (Tyr + HTyr) of 8 mg/day (with HP-EVOO) is even above the dose (5 mg/day) recommended by the EFSA to achieve beneficial health effects, including protection of LDL particles from oxidative damage and modulation of HDL levels [12,13]. Interestingly, our study did not reveal any significant differences in fasting plasma (poly)phenol levels, despite the substantial variation in (poly)phenol content among the three oils used. However, a trend toward increased levels was observed in participants supplemented with HP-EVOO, both in healthy individuals and post-MI patients. One possible explanation for this finding might be the rapid clearance of (poly)phenols from plasma. Previous studies have shown that the peak plasma concentration of HTyr metabolites occurs approximately 30 min after intake and declines sharply within 2 h post-administration [34,35]. In the present study, blood samples were collected after a 12 h fasting period, which likely reduced the likelihood of detecting peak levels. Additionally, the substantial inter-individual variability in phenolic compounds absorption, as previously reported [36,37], warrants consideration as well.

Olive oil was selected for this study, as it has been identified as the optimal source for Tyr/HTyr [36]. Similarly, Wood et al., reported no significant changes in fasting plasma total (poly)phenol levels after 12 weeks of wild blueberry supplementation (providing 302 mg of anthocyanins) in healthy older individuals [38]. However, they observed a significant increase in 24 h urinary excretion of (poly)phenol metabolites in the intervention group compared to the control group [38]. Unfortunately, our study did not include the collection of urinary samples from participants. In contrast, a randomized clinical trial in obese adults aged 40–65 years showed that replacing regular olive oil with an EVOO rich in oleocanthal and oleacein for 4 weeks significantly increased fasting plasma concentrations of HTyr in individuals with prediabetes and obesity [39].

The assessment of oxidative stress status is important, since it has been shown that ox-LDL plays a crucial role in the initiation of atherosclerotic plaque formation [40,41] and serves as a prognostic predictor in patients with chronic congestive heart failure [42]. Numerous in vitro and in vivo studies have demonstrated that (poly)phenols, particularly HTyr, exert cardioprotective effects by reducing oxidative stress and enhancing antioxidant biomarkers [10,43,44,45]. While our result showed no significant changes in plasma MDA levels, a marker of lipid oxidative stress, Fitó et al., reported a significant reduction in plasma lipid peroxide levels following the consumption of 50 mL/day of virgin olive oil for 3 weeks in patients with stable coronary heart disease [16]. Ikonomidis et al., also reported a significant decrease in MDA levels following a 4-week intervention in which patients took four capsules per day, each containing 412.5 mg of olive oil and 2.5 mg of HTyr, in patients with chronic coronary artery syndrome [46]. Consistently, Colica et al., demonstrated that the intake of two gastroresistant capsules containing 15 mg/day of HTyr for 3 weeks significantly reduced MDA levels in healthy individuals. They also showed that HTyr supplementation significantly increased the plasma HTyr, thiol groups, and total antioxidant status (TAS) [47]. However, the daily HTyr dose in their study (15 mg) was two times higher than that used in our own. In line with this, our study found that the HP-EVOO intervention for 26 weeks increased serum FRAP levels in post-MI individuals. Similarly, in a randomized crossover trial, short-term consumption of phenol-rich EVOO (69 g/day for 3 weeks) resulted in a slight, non-significant increase in FRAP in healthy individuals [48].

Regarding HDL functionality, a placebo-controlled intervention study found that co-supplementation of 9.9 mg of HTyr and 195 mg of punicalagin, taken three times per day for 8 weeks in healthy adults aged 45–65, did not lead to changes in ox-LDL, total plasma lipid peroxides, antioxidant capacity, or PON-1 levels [49]. In contrast, our study measured PON-1 activity rather than its concentration. Modulating PON-1 activity may serve as a promising therapeutic target to reduce the prevalence of atherosclerosis and its clinical complications [50]. PON-1 is an HDL-associated enzyme with antioxidant properties that help protect against oxidative stress [51]. Additionally, PON-1 has been shown to reduce inflammation by limiting LDL oxidation, thus playing a crucial role in preventing the initial stage of foam cell formation [52]. In our study, participants in the HP-EVOO group showed that serum PON-1 activity was significantly lower in post-MI patients compared to the healthy group at baseline. This finding is consistent with previous studies demonstrating that serum PON-1 activity is significantly reduced in patients with myocardial infarction, potentially contributing to the development of coronary artery disease [53]. Furthermore, the results of the present study indicate that the intake of 25 mL/day of HP-EVOO for 26 weeks significantly increased serum PON-1 activity in elderly post-MI patients. These findings suggest that regular consumption of EVOO, rich in (poly)phenols, particularly Tyr/HTyr, may enhance antioxidant status and help reduce the risk of recurrent CVD. This finding suggests that olive oil may exert cardiovascular protective effects not only through its polyphenol content—depending on their concentration—but also via its specific fatty acid composition. Oleic acid, the predominant monounsaturated fatty acid in olive oil, appears to positively influence PON-1 activity [54]. These observations are consistent with the study by Nguyen and Sok, which demonstrated that oleic acid protects PON-1 from oxidative inactivation and enhances its stability, in contrast to polyunsaturated fatty acids, which tend to inhibit its activity [55].

It is important to note that all post-MI participants were on stable statin therapy for 3 to 4 months prior to the beginning of the intervention and continued their use throughout the study period. Therefore, it is unlikely that statins alone accounted for the observed improvements in PON-1 activity and FRAP levels during the intervention.

Among the various enzymes involved in HDL remodeling, LCAT plays a crucial role. It facilitates HDL maturation by converting free cholesterol into cholesteryl esters, thereby promoting the transformation of nascent, discoidal HDL into a spherical, functionally active form [51]. Given this role, LCAT has been proposed as a potential therapeutic target for CVDs.

Farràs et al., reported that a 3-week intake of 25 mL/day of high-phenolic compound virgin olive oil (500 mg/kg) increased LCAT activity compared to low-phenolic compound virgin olive oil (80 mg/kg) in hypercholesterolemic individuals [56]. In contrast, our present study showed no significant change in LCAT activity following the intervention.

Although LCAT has long been considered a key enzyme in reverse cholesterol transport (RCT) and atheroprotection, studies in both animal models and humans have yielded conflicting results, likely due to unclear underlying mechanisms [57,58]. Elevated LCAT levels are typically associated with increased HDL-C concentrations; however, this does not consistently result in enhanced atheroprotective effects. Conversely, reduced LCAT levels and activity are often linked to lower HDL-C levels, yet this is not always accompanied by a greater extent of atherosclerosis [59]. These inconsistencies suggest that LCAT activity alone may not reliably reflect HDL functionality or its protective role in cardiovascular disease. Moreover, evidence indicates that cholesterol efflux from macrophages and RCT can still proceed even in the absence of functional LCAT, challenging earlier assumptions about its indispensable role [60].

Findings from the IMPROVE study, which included 540 European individuals at high cardiovascular risk, showed no association between plasma LCAT concentrations and carotid intima-media thickness (IMT), a marker of preclinical atherosclerosis, in the overall population, even after adjusting for age, sex, HDL-C, and triglycerides. These results further support the view that LCAT is not essential for effective RCT and that low LCAT levels are not necessarily linked to increased atherosclerosis risk [61].

Stadler et al., study showed that LCAT activity was not associated with the incidence of atherosclerotic cardiovascular events or kidney function decline during a 5-year follow-up in 453 non-dialysis CKD patients from the CARE FOR HOMe study. while low LCAT activity was independently associated with all-cause mortality and acute decompensated heart failure (ADHF) [62].

Altogether, these findings underscore that while LCAT plays a role in HDL metabolism, its impact on atherosclerosis is highly context-dependent and influenced by genetic, metabolic, and sex-specific factors. Thus, future preventative or therapeutic strategies should consider these complexities rather than relying solely on LCAT activity as a marker of cardiovascular protection.

An important factor to consider when interpreting the outcome results is that levels of total cholesterol, LDL-C, HDL-C, and non-HDL cholesterol were significantly higher in healthy participants compared to post-MI patients at both baseline and after the intervention within the HP-EVOO group. Similar results were observed in the EVOO group, except for HDL-C, which did not differ significantly between the two populations.

The first strength of this study lies in its comparison of three types of oils, with ROO serving as the control group. Secondly, instead of merely quantifying enzymes associated with HDL function, this study focused on measuring enzyme activity. A third strength is the examination of the long-term effects of consuming HP-EVOO compared to regular EVOO and ROO. Additionally, we selected oils from high-quality, technologically advanced products that possessed the specific characteristics necessary for this study. Moreover, the inclusion of both healthy elderly participants and post-MI patients allowed more comprehensive investigation of the effects. Finally, this study incorporated HTyr into the participants’ diet within the food matrix of EVOO, which has previously been shown to enhance HTyr bioavailability.

Nevertheless, the present study has certain limitations. One limitation was the relatively small sample size, which may have reduced the statistical power to detect differences in biomarkers with high interindividual variability in some groups. Another limitation was the lack of assessment of physical activity or other lifestyle factors, which could have influenced the results. Additionally, the study did not examine mechanistic outcomes such as changes in gut microbiota, cellular signaling pathways, gene expression, or enzyme activities related to cardiovascular health. These aspects should be explored in future research.

## 5. Conclusions

Overall, this pilot study demonstrated that consuming HP-EVOO may have beneficial effects on the antioxidant status of patients with a history of myocardial infarction, such as FRAP and PON-1 activity. Although the cardiovascular protective effects of HTyr have been documented, further long-term clinical studies involving diverse populations are needed. These studies should account for environmental, lifestyle, and genetic variations to define more clearly the specific effects of HTyr on oxidative stress and HDL functionality. Notably, HDL functionality and the distribution of its subclasses have been shown to be more important than HDL quantity in the prevention of CVD.

## Figures and Tables

**Table 1:** Baseline characteristics of the participants (data are presented as numbers or mean ± SE).

		HP-EVOO			EVOO			ROO		
		Healthy	Post-MI	*p*-Value (Between Groups)	Healthy	Post-MI	*p*-Value (Between Groups)	Healthy	Post-MI	*p*-Value (Between Groups)
Sex	Women	1	1		3	2		4	1	
	Men	4	6		3	5		2	2	
Age		71 ± 2.34	75.71 ± 2.97	0.272	76.67 ± 1.82	71.5 ± 2.19	0.124	76 ± 2.87	73.67 ± 2.33	0.618
Weight (kg)	Before	94 ± 15.70	79.79 ± 4.66	0.339	68.85 ± 7.37	73.13 ± 3.15	0.584	78.78 ± 5.34	81.73 ± 14.09	0.814
	After	95.62 ± 16.82	80.58 ± 4.98	0.376	69.32 ± 6.90	75.96 ± 2.98	0.433	81.83 ± 4.10	82.83 ± 13.06	0.714
*p*-value (Within groups)		0.300	0.062		0.473	0.909		0.156	0.641	
BMI (kg/m^2^)	Before	31.51 ± 4.85	26.58 ± 1.06	0.431	25.70 ± 2.00	27.15 ± 1.08	0.521	28.47 ± 1.86	28.33 ± 2.75	0.969
	After	32.05 ± 5.20	27.08 ± 1.19	0.536	25.93 ± 1.88	28.09 ± 1.274	0.536	29.61 ± 1.60	28.97 ± 2.48	0.828
*p*-value (Within groups)		0.307	0.062		0.385	0.812		0.184	0.318	
Systolic BP (mmHg)	Before	143.4 ± 6.76	147.0 ± 9.27	0.778	136.4 ± 6.50	131.1 ± 2.50	0.413	146.0 ± 9.55	132.0 ± 1.52	0.351
	After	144.4 ± 5.51	126.0 ± 9.15	0.137	124.3 ± 11.83	137.0 ± 7.50	0.412	141.8 ± 12.38	136.3 ± 12.12	0.789
*p*-value (Within groups)		0.731	0.051		0.366	0.589		0.539	0.730	
Diastolic BP (mmHg)	Before	82.60 ± 1.36	76.14 ± 1.80	**0.024**	80.20 ± 11.18	79.57 ± 2.25	0.406	81.50 ± 2.66	78.33 ± 2.66	0.357
	After	84.40 ± 1.36	79.50 ± 3.87	0.299	74.50 ± 3.83	81.00 ± 5.814	0.684	80.33 ± 4.74	78.33 ± 6.36	0.812
*p*-value (Within groups)		0.321	0.328		>0.999	0.812		0.672	>0.999	
CRP (mg/L)	Before	2.60 ± 1.10	2.957 ± 1.31	0.621	1.95 ± 0.52	1.14 ± 0.19	0.188	1.13 ± 0.24	1.63 ± 0.73	0.690
	After	2.48 ± 1.65	1.817 ± 0.48	0.541	2.23 ± 0.58	1.15 ± 0.22	0.162	1.20 ± 0.18	0.70 ± 0.10	0.059
*p*-value (Within groups)		> 0.999	>0.999		0.377	0.500		0.562	0.500	
TC (mmol/L)	Before	4.89 ± 0.29	3.12 ± 0.22	**0.0007**	5.07 ± 0.25	3.15 ± 0.19	**0.0003**	4.71 ± 0.27	3.09 ± 0.09	0.0054
	After	4.89 ± 0.29	3.30 ± 0.27	**0.003**	5.375 ± 0.18	3.08 ± 0.16	**<0.0001**	4.81 ± 0.49	3.05 ± 0.11	**0.047**
*p*-value (Within groups)		0.991	0.575		0.179	0.654		0.739	0.269	
TG (mmol/L)	Before	1.11 ± 0.35	1.21 ± 0.26	>0.999	1.38 ± 0.22	1.15 ± 0.35	0.400	0.88 ± 0.13	0.87 ± 0.07	0.956
	After	0.95 ± 0.17	1.62 ± 0.48	0.258	1.45 ± 0.31	0.84 ± 0.061	0.063	0.98 ± 0.10	0.85 ± 0.10	0.479
*p*-value (Within groups)		0.437	0.313		0.710	0.812		0.528	0.431	
HDL-C (mmol/L)	Before	1.72 ± 0.23	1.13 ± 0.11	**0.031**	1.59 ± 0.25	1.18 ± 0.11	0.202	1.69 ± 0.15	1.28 ± 0.08	0.125
	After	1.79 ± 0.24	1.13 ± 0.16	**0.048**	1.68 ± 0.27	1.27 ± 0.09	0.167	1.69 ± 0.18	1.390 ± 0.09	0.316
*p*-value (Within groups)		0.380	0.486		0.082	0.425		0.957	0.085	
LDL-C (mmol/L)	Before	2.66 ± 0.31	1.43 ± 0.24	**0.010**	2.84 ± 2.84	1.44 ± 0.10	**0.0001**	2.61 ± 0.21	1.41 ± 0.03	**0.006**
	After	2.66 ± 0.26	1.42 ± 0.24	**0.007**	3.02 ± 0.11	1.42 ± 0.10	**<0.0001**	2.68 ± 0.35	1.27 ± 0.07	**0.030**
*p*-value (Within groups)		0.961	0.724		0.344	0.897		0.765	0.063	
non-HDL-C (mmol/L)	Before	3.07 ± 0.42	1.99 ± 0.23	**0.035**	3.47 ± 0.23	1.92 ± 0.13	**0.002**	3.01 ± 0.18	1.81 ± 0.01	**0.002**
	After	3.10 ± 0.26	2.17 ± 0.18	**0.016**	3.68 ± 0.17	1.80 ± 0.14	**<0.0001**	3.12 ± 0.34	1.66 ± 0.037	**0.022**
*p*-value (Within groups)		0.668	0.665		0.312	0.602		0.671	**0.021**	
Lp (a) (nmol/L)	Before	32.56 ± 10.69	71.55 ± 34.68	0.349	52.82 ± 45.12	72.06 ± 29.37	0.190	114.1 ± 29.97	141.4 ± 51.41	0.635
	After	31.56 ± 10.52	89.67 ± 40.08	0.231	60.82 ± 50.50	104.1 ± 43.38	0.142	107.0 ± 24.09	81.00 ± 13.23	0.497
*p*-value (Within groups)		0.228	0.353		0.250	0.437		0.601	0.263	

Bold *p*-values indicate statistical significance (*p* < 0.05). Abbreviations: BMI, body mass index; BP, blood pressure; HDL-C, high-density lipoprotein cholesterol; LDL-C, low-density lipoprotein cholesterol; Lp (a), lipoprotein(a); TC, total cholesterol; TG, triglycerides.

## Data Availability

Data are contained within the article.

## Abbreviations

ASCVD	Atherosclerotic cardiovascular disease
BMI	Body mass index
BP	Blood pressure
CRP	C-reactive protein
CVD	Cardiovascular disease
EFSA	European Food Safety Authority
EVOO	Extra virgin olive oil
FRAP	Ferric reducing antioxidant power
HDL	High-density lipoprotein
HO-1	Heme oxygenase-1
HP-EVOO	High-phenolic (HTyr/Tyr) extra virgin olive oil
HTyr	Hydroxytyrosol
IHD	Ischemic heart disease
LCAT	Lecithin-cholesterol acyltransferase
LDL	Low-density lipoprotein
Lipoprotein (a)	Lp (a)
LPS	Lipopolysaccharide
MD	Mediterranean diet
MDA	Malondialdehyde
MUFA	Monounsaturated fats
NO	Nitric oxide
Nrf2	Nuclear factor erythroid 2-related factor 2
ox-LDL	Oxidized low-density lipoprotein
PON-1	Paraoxonase-1
Post-MI	Post-myocardial infarction
ROO	Refined olive oil
ROS	Reactive oxygen species
SE	Standard error
SOD-1	Superoxide dismutase-1
TAC	Total antioxidant status
TC	Total cholesterol
TG	Triglyceride
Tyr	Tyrosol
